# Expression of concern for Euro Surveill. 2026;31(2)

**DOI:** 10.2807/1560-7917.ES.2020.31.3.260122ec

**Published:** 2026-01-22

**Authors:** Eurosurveillance editorial team

**Affiliations:** 1 European Centre for Disease Prevention and Control (ECDC), Stockholm, Sweden

Regarding the article "Moderate protection from vaccination against influenza A(H3N2) subclade K in Beijing, China, September to December 2025" by Shen et al, published on 15 January 2026, the corresponding author has informed *Eurosurveillance* of a data handling error in the inclusion of influenza-like illness cases which may have led to biased influenza vaccine effectiveness estimates. We are currently investigating the situation related to this honest error and will take further steps as necessary. 

While we await more detailed results, the editors decided to publish an expression of concern for this article. We are grateful to the authors for flagging the issue in a timely manner.

